# Seroprevalence of SARS‐CoV‐2 Antibody Before and After Both Vaccination and Natural Infection in China

**DOI:** 10.1002/iid3.70184

**Published:** 2025-03-24

**Authors:** Yang Guang, Liu Lina, Liu Hui

**Affiliations:** ^1^ Department of Laboratory and Quarantine Dalian Medical University Dalian Liaoning China

**Keywords:** antibody, COVID‐19, public health, SARS‐CoV‐2, vaccination

## Abstract

**Objective:**

This study aims to analyze the effects of temporal SARS‐CoV‐2 antibodies in China before and after both vaccination and natural infection, thereby providing an empirical basis for evaluating the effectiveness of various prevention methods, including vaccination.

**Methods:**

IgG antibodies against SARS‐CoV‐2 were determined using chemiluminescence immunoassays, and antibody data was collected from published articles starting in early 2020 and from patients scheduled for surgery at the Hospital of Dalian Medical University between January 2022 and January 2024.

**Results:**

A SARS‐CoV‐2 infection epidemic in Wuhan in January 2020 led to a 3.2% seropositivity rate of SARS‐CoV‐2 antibodies (total antibodies). While the seropositivity rate for SARS‐CoV‐2 antibodies in mainland China reached 37.2% following the implementation of China's zero‐COVID policy and the immunization rate was above 90% in January 2022. By the end of 2022, the Chinese government eased strict control measures, resulting in a SARS‐CoV‐2 antibody (IgG) positivity rate of 86.7% in January 2023. In January 2024, the positivity rate for SARS‐CoV‐2 antibodies in post‐pandemic was recorded at 94.0%. Antibody levels in the early part of 2023 were considerably higher than those measured in January 2022 (68.66 vs. 10.21, *p* < 0.05); that in early 2024 were not substantially higher than those in January 2023 (49.29 vs. 68.66, *p* > 0.05).

**Conclusions:**

The results of this study indicated that the immune barrier established by inactivated vaccines could be disrupted by the natural infection with SARS‐CoV‐2, resulting in a higher level of antibody production than vaccination. This effect can last for more than a year.

## Graphical abstract

This study analyzes the levels of antibodies to SARS‐CoV‐2 at key time points during the COVID‐19 pandemic in China, before and after the impact of vaccination on antibodies and the level of antibodies during natural infection after relaxed strict control measures to provide a scientific basis to study the prevalence of COVID‐19 and evaluate various preventive and control measures.

## 1 Introduction

The severe acute respiratory syndrome coronavirus 2 (SARS‐CoV‐2), responsible for Coronavirus Disease 2019 (COVID‐19), poses a considerable global public health threat. This virus predominantly exists in the respiratory secretions of infected individuals and is disseminated via respiratory droplets released during actions like sneezing and coughing [1]. Most people recover when the adaptive immune response is activated, which results in the production of particular antibodies and other immunological components. Immune cells in the alveoli, such as macrophages, target and destroy the infected cells and phagocytose the cellular debris.

SARS‐CoV‐2 is a novel virus, resulting in a lack of pre‐existing immunity in the population before 2019. On December 8, 2019, pneumonia of unknown origin, subsequently recognized as COVID‐19, was initially detected in Wuhan, China [2]. As of January 30, 2020, China reported a mortality rate of 2.1%, with 12,050 confirmed cases of COVID‐19 and 259 deaths [3]. Mainland on January 20, 2020, China implemented strict preventive and control measures, precisely a “zero‐COVID” policy. Post‐April 2020, the pandemic was predominantly managed, with only a limited number of isolated cases persisting [3].

Following successful development and production, COVID‐19 vaccines were widely distributed in 2021. By mid‐2021, the vaccination rate for virus‐inactivated vaccines in mainland China reached 90% [4]. Despite this high coverage, mainland China maintained strict “zero‐COVID” measures until 2022. The pandemic peaked at the end of 2022 and early 2023, reaching its highest point at the end of January 2023 after the relaxation of stringent control measures [4, 5]. Preventive and control measures implemented during the pandemic including vaccination and nonpharmacological alternatives is a significant case for analyzing the evolution of the pandemic and assessing the efficacy of the measures enacted.

There have been many reports about seroprevalence of SARS‐CoV‐2 antibody during and after the COVID‐19 pandemic [6, 7, 8]. However, most studies on anti‐SARS‐CoV‐2 antibodies are from outside of China [9, 10, 11], where the Chinese government's control measures have not been implemented. Thus, several factors influence the accuracy of the findings. Therefore, clean data with minimal background interference may be obtained by monitoring anti‐SARS‐CoV‐2 antibodies on the Chinese mainland. This study evaluates real‐world data on SARS‐CoV‐2 antibody levels collected at key times during the COVID‐19 pandemic and before and after COVID‐19 vaccination to determine the efficacy of different preventive and control measures.

## 2 Materials and Methods

### 2.1 Study Design

This study used data from the start of the COVID‐19 pandemic (2020) from published literature to continuously monitor SARS‐CoV‐2 antibody levels in the population before and after COVID‐19 vaccination, as well as at crucial points during the pandemic. Data from pre‐natural infection (2022), post‐natural infection (2023), and after 1 year from natural infection (2024) were derived from direct measurements of antibody levels.

### 2.2 Data Collection for 2020

The PubMed database was searched for information on SARS‐CoV‐2 IgG antibody positivity rates, and relevant data from early 2020 were gathered from published literature to meet the following inclusion criteria: (1) original study; (2) selection of healthy subjects; (3) investigation of more than 10,000 subjects; and (4) data obtained in early 2020. A total of four articles were included [12, 13, 14, 15]. The exclusion criteria were the selection of high‐risk populations, such as medical workers, which led to the exclusion of one article [15].

### 2.3 Data Collection After 2021

Direct measurements of antibody levels provided the data from January 2022 to January 2024. Serum samples taken from inpatients at Dalian Medical University's Second Hospital were used to assess these antibody levels. All patients had tested negative for COVID‐19 within 1 week before the sample collection and had received at least 1 dose of the COVID‐19 vaccine. The exclusion criteria comprised samples lacking medical records or partial records. The medical records of all patients who submitted sera were carefully reviewed to confirm compliance with all inclusion criteria. No participants were excluded from the study.

The average age of patients in the January 2022 group was 56.2 ± 19.2 years, consisting of 78 males and 59 females. The January 2023 group had a mean age of 56.7 ± 19.0 years, comprising 57 males and 78 females. The same demographics applied to the January 2024 cohort, with a mean age of 50.7 ± 20.1 years and 30 males and 20 females.

### 2.4 Serological Assay

Antibody levels (IgG) were measured using a chemiluminescence immunoassay (S/CO), with a threshold value exceeding 1.00 deemed positive for antibody presence. Non‐normally distributed data were represented as medians and interquartile ranges. The chemiluminescence immunoassay (AutoLumoA2000Plus) was conducted in the clinical laboratory of our University Hospital employing standard commercial reagent kits. The coefficient of intra‐assay variation was below 5%.

### 2.5 Statistical Analysis

The Mann–Whitney test was employed to assess significant differences between groups, given the abnormal distribution of the antibody data. The significance threshold was set at *p* ≤ 0.05 (two‐sided). Statistical analyses were conducted using SPSS statistical analysis software for Windows with default settings.

## 3 Results and Discussion

### 3.1 Begin in the COVID‐19 Pandemic

Favorable rates of SARS‐CoV‐2 antibodies from early 2020 were extracted from the literature [12, 13, 14] and, respectively, 2.4%, 3.2%, and 5.6% (said 3.2% with median). SARS‐CoV‐2 was first identified in Wuhan, China, in 2019. Before the outbreak, no SARS‐CoV‐2 antibodies were found in the public because SARS‐CoV‐2 was a novel virus. Because there was currently no effective vaccination, the results might suggest that up to 3.2% of the population had contracted the viral infection.

### 3.2 Before and After Vaccination

**Figure 1:**
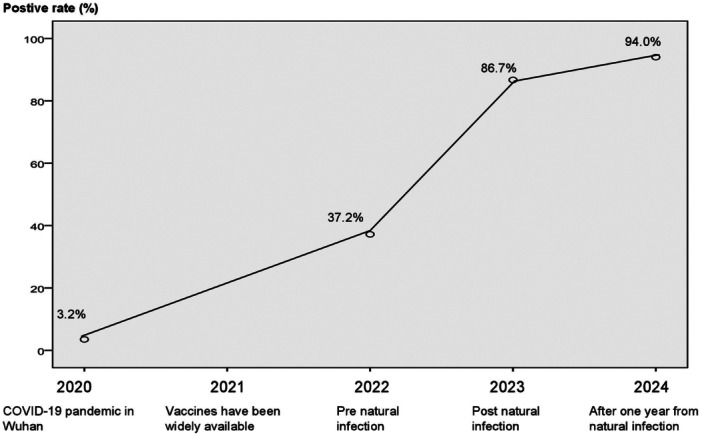
Positivity rates of antibodies to SARS‐CoV‐2 at critical time points before and after the COVID‐19 pandemic in China.

In mainland China, the immunization rate reached 90% in 2021 following the successful introduction of vaccinations at the end of 2020 [4]. Direct assessments of antibody levels from collected samples were used to determine the levels of antibodies to SARS‐CoV‐2. The antibody‐positive rate in the Dalian population was 37.2% in the January 2022 cohort. This was a significant increase relative to the pre‐vaccination period when the positive rate of antibodies in China was 3.2%, as depicted in Figure 1. The strict preventive and control measures, along with the “zero‐COVID” policy implemented by Mainland China from January 2020 to December 2022, suggest that the elevated antibody positivity rate in January 2022 was predominantly attributable to vaccination rather than spontaneous infection.

### 3.3 Nature Infection

COVID‐19 peaked in early 2023 following the Chinese government's relaxation of stringent control measures and adoption of normalized prevention strategies at the end of 2022. The January 2023 cohort had an antibody‐positive rate of 86.7%, suggesting that natural infection played a significant role in the elevated antibody levels.

Moreover, a quantitative antibody assay was performed on individuals with positive antibody results. The results revealed that antibody levels in early 2023 were considerably higher than those measured in January 2022 (*p* < 0.05), as shown in Table 1. The results also showed that the elevated antibody levels seen in early 2023 were primarily due to natural infection, as vaccinations made from inactivated viruses may be less effective [16].

**Table 1:** Levels of anti‐SARS‐CoV‐2 antibodies in the population with antibody positivity.

Times	Quartile			*z* value	*p* value
	25th	50th	75th		
Pre‐natural infection (January 2022)	4.520	10.210	25.260	13.785	< 0.001
Post‐natural infection (January 2023)	7.773	68.660	152.970		
Post‐natural infection (January 2023)	7.773	68.660	152.970	0.898	0.369
One year after the natural infection	20.66	49.29	75.82		

### 3.4 After Nature Infection

Following a year of natural infection, the January 2024 cohort had the same demographics; Figure 1 illustrates the 94.2% antibody‐positive rate. Furthermore, Table 1 showed that there was no significant difference in antibody titers between January 2023 and early 2024 (*p* > 0.05). This implies that the majority of the antibodies against the virus may have come from the original natural infections and may have lasted more than a year in naturally infected people.

Vaccines based on inactivated viruses can be less effective [16], the present data also showed that the positive rate of antibodies was not very high in the real world; therefore, they do not interfere significantly with antibodies produced in response to natural infection. This is one of the main limitations of this study. The observed antibody positivity rate (86.7%) in the population does not completely rule out the contribution of vaccination. Another limitation is that this study did not measure cellular immunity and neutralization. Therefore, the protective effect of vaccination and natural infection needs further evaluation.

In conclusion, the results show that natural SARS‐CoV‐2 infection produced more antibodies than vaccination and that the immune barrier established by inactivated vaccines could be disrupted by the natural infection. The production of antibodies is dependent on the process of virus proliferation in the body, and natural SARS‐CoV‐2 infection produces high antibody levels that can last for more than a year.

## Author Contributions

Yang Guang conceived the analysis and wrote the final version of the manuscript. Liu Lina provided technical support and data collection. Liu Hui conceived the analysis and critically revised the manuscript.

## Ethics Statement

The Dalian Medical University Ethics Committee approved the study without informed consent, as the samples were remnants following clinical use and not specifically collected for this study and were therefore without risk to the patients.

## Data Availability

The data that support the findings of this study are available on request from the corresponding author.
